# Anodal Stimulation: An Underrecognized Cause of Nonresponders to Cardiac Resynchronization Therapy

**Published:** 2011-05-01

**Authors:** Khalin F Dendy, Brian D Powell, Yong-Mei Cha, Raul E Espinosa, Paul A Friedman, Robert F Rea, David L Hayes, Margaret M Redfield, Samuel J Asirvatham

**Affiliations:** 1 Department of Internal Medicine; Mayo Clinic, Rochester, Minnesota; 2 Division of Cardiovascular Diseases; Mayo Clinic, Rochester, Minnesota

**Keywords:** Cardiac resynchronization therapy, anodal stimulation, biventricular pacemaker, heart failure

## Abstract

**Objective:**

The purpose of this study was to determine if anodal stimulation accounts for failure to benefit from cardiac resynchronization therapy (CRT) in some patients.

**Background:**

Approximately 30-40% of patients with moderate to severe heart failure do not have symptomatic nor echocardiographic improvement in cardiac function following CRT. Modern CRT devices allow the option of programming left ventricular (LV) lead pacing as LV tip to right ventricular (RV) lead coil to potentially improve pacing thresholds. However, anodal stimulation can result in unintentional RV pacing (anode) instead of LV pacing (cathode).

**Methods:**

Patients enrolled in our center's CRT registry had an echocardiogram, 6-minute walk (6MW), and Minnesota Living with HF Questionnaire (MLHFQ) pre-implant and 6 months after CRT. Electrocardiograms (12 lead) during RV, LV, and biventricular (BiV) pacing were obtained at the end of the implant in 102 patients.  Anodal stimulation was defined as LV pacing QRS morphology on EKG being identical to RV pacing or consistent with fusion with RV and LV electrode capture. LV end systolic volume (LVESV) was measured by echo biplane Simpson's method and CRT responder was defined as 15% or greater reduction in LVESV.

**Results:**

Of the 102 patients, 46 (45.1%) had the final LV lead pacing configuration programmed LV (tip or ring) to RV (coil or ring).  3 of the 46 subjects (6.5%) had EKG findings consistent with anodal stimulation, not corrected intraoperatively.  All anodal stimulation patients were nonresponders to CRT by echo criteria (reduction in LVESV 13.3 ± 0.6%, increase in EF 5.0 ± 1.4%) compared to 46% responders for those without anodal stimulation, (change in LVESV 18.7 ± 25.6%, EF 7.6 ±10.9%). None of the anodal stimulation patients were responders for the 6 minute walk, compared to 32 of 66 (48%) of those without anodal stimulation.

**Conclusion:**

Anodal stimulation is a potential underrecognized and ameliorable cause of poor response to CRT.

## Introduction

Heart failure is a significant cause of morbidity and mortality affecting nearly five million people in the United States, with approximately 500,000 newly diagnosed cases each year [1,2]. Cardiac resynchronization therapy (CRT) has emerged as a means to improve quality of life, reduce hospitalizations, and increase survival through reversal of left ventricular remodeling and slowing of disease progression in patients with heart failure, reduced left ventricular ejection fraction (LVEF), and a prolonged QRS duration [3-5]. However, approximately 30-40% of patients with moderate to severe heart failure do not have symptomatic nor echocardiographic improvements in cardiac function following CRT [6,7]. Failure of studies to provide a unifying cause of nonresponse to CRT has suggested a multifactorial etiology. We hypothesized that anodal stimulation is one of the etiologies of nonresponse.

**Figure 1:**
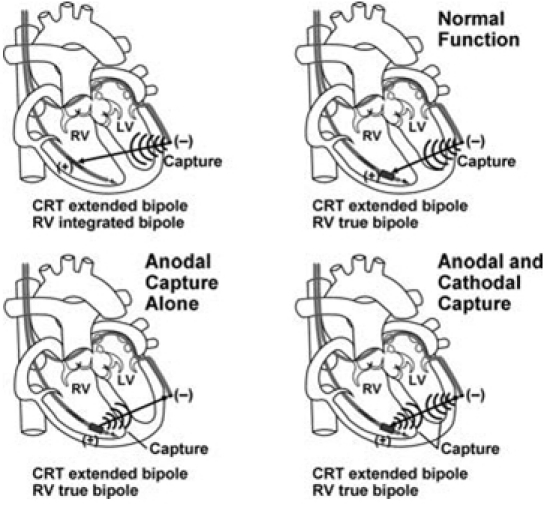
The upper two figures demonstrate normal cathode capture in the setting of integrated and dedicated bipolar RV leads.  The lower left figure is an illustration of attempted pacing from the LV electrode resulting instead in anodal stimulation of the RV ring electrode. The lower right figure illustrates capture of both the cathode (LV electrode) and anode (RV ring electrode) resulting in fusion.  Reproduced with permission (CD Swerdlow, et al. PACE 2006; 29(1): 70-96)

Standard bipolar pacing is between two closely spaced electrodes, such as left ventricular (LV) lead tip electrode to LV ring electrode. Capture typically occurs at the cathode. Modern CRT devices allow the option of programming LV lead pacing as LV tip electrode (cathode) to right ventricular (RV) lead coil or ring (anode) as an alternative to improve pacing thresholds. Anodal stimulation (Figure 1) is defined as capture at the pacing anode instead of cathode [8,9]. This is more common at higher pacing outputs. If anodal stimulation occurs when a CRT device is programmed LV tip to RV coil, the RV is unintentionally captured instead of the LV. If not identified and corrected at the time of CRT implant, cardiac resynchronization will not occur. The objective of this study was to determine if anodal stimulation accounts for failure to benefit from CRT in some patients.

## Methods

### Patient Population

Patients who underwent clinically indicated biventricular pacemaker implantation (with or without defibrillator) at our institution between September 2005 and October 2007 were offered the opportunity to enroll in the cardiac resynchronization therapy registry. Of the 185 patients enrolled in the registry, there were 102 patients with comprehensive RV, LV, and BiV pacing EKG data which were included in this study. The study was approved by the Institutional Review Board.

### CRT Therapy

Commercially available CRT devices and leads were used. Standard settings included atrioventricular delay of 100 ms (sensed) and 130 ms (paced), interventricular delay of 0 ms with DDD or DDDR mode and standard lower (50-60 beats/min) and upper (120-130 beats/min) pacing rates. At the time of CRT implant, LV lead thresholds were tested LV tip to LV ring, and LV tip or ring to RV coil or ring, depending on the CRT device pacing configuration options. The final programmed setting was the LV pacing configuration that resulted in the lowest pacing threshold.

### Baseline and Follow-up Evaluation

This single center registry of prospectively collected data included baseline characteristics, extensive echocardiography data, device implant data, and lead position data.  Patient data was collected prior to CRT implantation and at 6 month follow-up.  At baseline and the time of each follow-up, patients underwent laboratory testing, comprehensive echocardiograms, device interrogation, 6 minute walk, oxygen consumption exercise treadmill testing, completion of Minnesota Living with Heart Failure Questionnaire, and evaluation of NYHA class. Left ventricular end systolic volume (LVESV) and left ventricular ejection fraction (LVEF) were measured by echo biplane Simpson's method [6,10].  A CRT responder for each separate endpoint was defined as 15% or greater reduction in LVESV, 10% or greater increase in EF, 10% or greater increase in 6-minute walk distance, 10% or greater reduction from baseline in MLHFQ, or any NYHA class improvement [11].

### Electrocardiography

Electrocardiograms (12 lead) were obtained on a digital recording system at an increased sweep speed of 100 mm/sec during LV, RV, and biventricular (BiV) pacing at the end of CRT implant. All tracings were reviewed independently by 2 physicians (KDF and BDP). Anodal stimulation was defined as LV pacing QRS morphology on EKG being consistent with partial or complete RV coil or ring capture.  This was defined as LV pacing either 1. Being identical to RV pacing QRS morphology, or 2. Fusion of RV and LV electrode capture based on LV pacing QRS vectors being inconsistent with anticipated EKG morphology for an LV lead pacing location (ie. LV pacing resulting in a positive QRS vector in lead I, negative in V1, or negative in leads II, III, and aVF if the LV lead was in an anterior or anterolateral location).  Chest x-rays were reviewed for LV lead position to exclude closely placed LV and RV leads (within 2 cm) as the reason for the same EKG morphology.

## Results

Of the 102 patients, 46 (45.1%) had the final LV lead pacing configuration programmed LV (tip or ring) to RV (coil or ring). Three of the 46 subjects (6.5%) were confirmed to have anodal stimulation, not corrected intraoperatively. 

Baseline characteristics showed the anodal stimulation group to have a higher rate of ischemic cardiomyopathy, higher LVESV and 6MW, and a lower LVEF.  Full Baseline characteristics are listed in Table 1.  No patients in the anodal stimulation group met the definition of responder in LVESV, LVEF, or 6MW.  Full details of response to CRT at six months are shown in Table 2.

**Figure 2:**
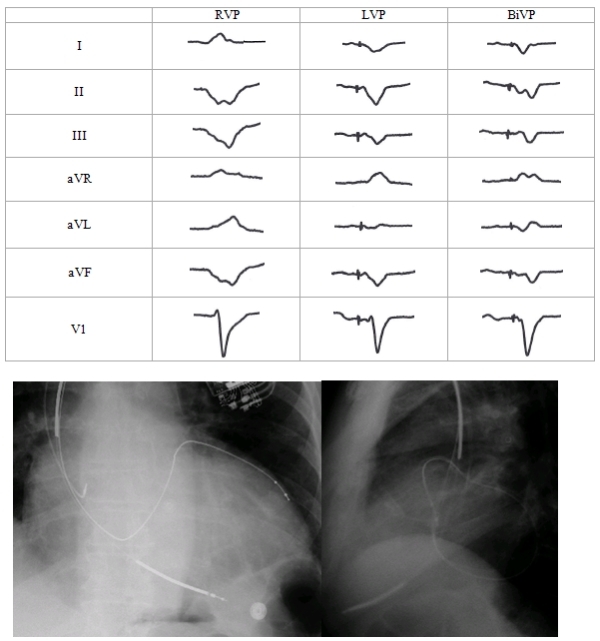


Anodal stimulation patient #1 was an 81 year old male with ischemic cardiomyopathy (LVEF 16%), QRS duration of 186ms, and NYHA class III functional status on maximal medical therapy.  A Guidant H170 CRT-D pulse generator was placed. He had a Medtronic 6949 Sprint Fidelis RV defibrillator lead positioned in the RV apex. The LV lead (Boston Scientific Easy Track 2) was placed in an anterolateral vein branch of the coronary sinus. LV lead was programmed LV ring to RV coil at a pacing output of 2.6 Volts, 0.50 msec. Lead position and EKG tracings are shown in Figure 2.  In spite of the LV lead being in an anterolateral location, LV pacing demonstrated a negative QRS vector in leads II, III, and aVF, which was similar to RV pacing.  Capture at the location of the LV lead ring would be expected to result in a positive QRS in II, III, and aVF. Lead I was negative, as would be expected from the LV lead tip pacing location. This patient demonstrated anodal stimulation with capture at both the anode (RV coil) and cathode (LV lead tip). LV pacing QRS morphology was similar to BiV pacing. At 6 month follow-up LVESV decreased from 287ml to 250ml, EF increased from 14% to 20%, NYHA class decreased from III to II.

**Figure 3:**
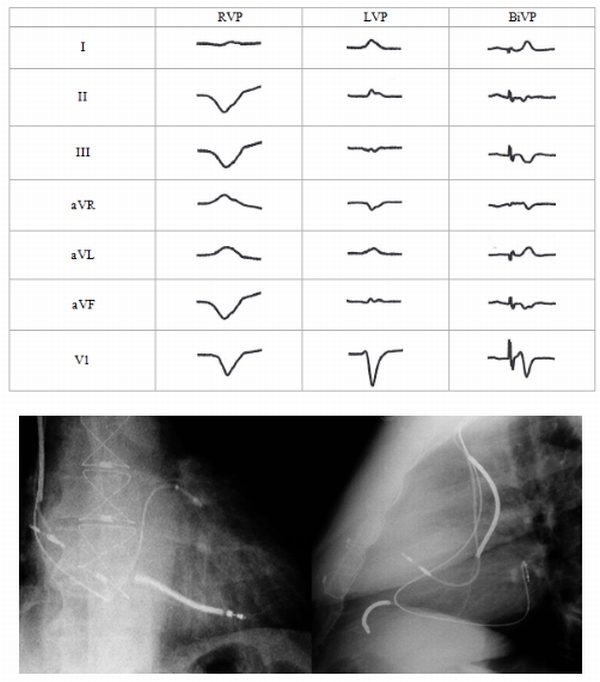


Anodal stimulation patient #2 was a 68 year old male with ischemic cardiomyopathy (LVEF 23%), QRS duration 138ms, and NYHA class III functional status on maximal medical therapy. A Medtronic 7991 ID pulse generator was placed.  He had a Medtronic 6947 Sprint Quattro Secure RV defibrillator lead positioned in the RV apex. The LV lead (Boston Scientific Easy Track 2) was positioned in the basal lateral location.  LV lead minimum threshold after testing various pacing configurations was 2.4 Volts at 0.50 msec and was with pacing LV tip to RV coil.  The final LV pacing output was programmed LV tip to RV coil at 5.0 Volts, 0.50 msec. Lead position and EKG tracings are shown in Figure 3.  Pacing from this location would be expected to have a negative QRS complex in lead I and positive in V1.  In this patient, there was capture of the anode (RV coil) during LV pacing, resulting in positive QRS in lead I, predominantly negative QRS in V1 (left bundle branch block type of pattern), and positive in aVL.  The QRS complex was also more narrow than typical for pacing the far lateral LV wall (LV pacing QRS duration = 143msec, RV pacing QRS = 143 msec, BiV pacing QRS = 137 msec).  The narrow QRS could have been due to capture of the RV septum, or capture at both the cathode (LV tip) and anode (RV coil) as seen in fused anodal stimulation and shown in Figure 1.  At 6 month follow-up LVESV decreased from 153ml to 132ml, EF increased from 24% to 28%, NYHA class was unchanged at III, 6MW slightly decreased from 430m to 419m, and MLWHF score was unchanged from 65 to 66.

Anodal stimulation patient #3 was a 61 year old male with ischemic cardiomyopathy (LVEF 19%), QRS duration 96ms, and NYHA class II functional status.  He required pacemaker placement following planned AV node ablation for symptomatic atrial fibrillation.  Although his pre-ablation QRS was narrow he underwent CRT-D placement as it was known he would have post-ablation continuous ventricular pacing in the setting of a low LVEF and heart failure.  A Guidant H217 pulse generator was placed. A Medtronic 6949 Sprint Fidelis lead was positioned in the RV apex.  A Boston Scientific Easy Track 2 lead was placed in the anterior interventricular vein after inadequate thresholds and/or phrenic nerve stimulation were found at more lateral sites.  LV lead was programmed LV tip to RV coil at a pacing output of 5.0 Volts, 0.50 msec.  In spite of LV pacing from an anterior location, the QRS vector was negative in leads II, III, and aVF, consistent with anodal stimulation. At 6 month follow-up EF was slightly decreased from 26% to 22%, NYHA class was unchanged at III, 6MW decreased from 530m to 476m, and MLWHF score increased from 16 to 28.

## Discussion

The main findings from this study are the following:  1. Anodal stimulation is underrecognized and was found in 6.5% of patients programmed LV tip to RV coil or ring, 2. Patients with anodal stimulation have a decreased response to CRT.

### Overall CRT Response

Past studies have reported a 60-70% response rate to CRT [3-5]. The lack of a unifying cause of nonresponse to CRT has led to a belief in multifactorial etiology. Prior studies have proposed etiologies that include myocardial scar burden [12], suboptimal LV lead position [13], and suboptimal interventricular pacing delay [14].  We have found anodal stimulation as an additional cause of poor response to CRT.

### Anodal stimulation is underrecognized

The incidence of anodal stimulation has not been documented in prior CRT studies. Anodal stimulation was recognized and corrected intraoperatively in some patients included in this study's control group. In spite of that, anodal stimulation went unrecognized in 6.5% of CRT implants with LV pacing configuration of LV to RV in this study.  Pacing LV tip or ring to RV coil or ring is often used due to lower pacing thresholds in some patients (45% of this study population). These pacing configurations can lead to clinically significant anodal stimulation resulting in RV capture during attempted LV pacing.

### Decreased response to CRT when anodal stimulation present

Poor CRT response when anodal stimulation is present is consistent with physiologic principles of ventricular synchrony. The benefit from CRT is believed to occur through improvement in left ventricular late depolarization in patients with left bundle branch block [15]. CRT with anodal stimulation essentially results in RV pacing alone, leading to persistent interventricular and intraventricular conduction delay to the LV lateral wall.

### Risk factors for anodal stimulation

Anodal stimulation is more likely to occur if:

1. Pacing from an LV lead electrode to a dedicated bipolar RV lead.

2. Pacing at high outputs.

3. Clinically relevant if pacing from an LV electrode to a distant RV coil or ring.

Prior studies have suggested that anodal stimulation is more common in dedicated than integrated bipolar defibrillation leads [8,16]. The increased incidence of anodal stimulation in dedicated bipolar leads has been suggested to be due to smaller lead surface area resulting in higher current density causing increased risk of surrounding myocardial depolarization. All three anodal stimulation patients we report had dedicated bipolar RV leads, but had the LV pacing configuration programmed LV tip to RV coil (as opposed to RV ring). Pacing configurations from an LV electrode to the RV ring are at greater risk for anodal stimulation; however anodal stimulation can occur with pacing from the LV electrode to either the RV ring or coil.

### Study Limitations

The incidence of anodal stimulation that was corrected at the time of device implant is not known.  This study demonstrated the incidence of undetected anodal stimulation and subsequent patient outcomes. Some patients were excluded due to incomplete follow-up data. Response rates of the overall CRT group are lower than some other reported trials. This may be secondary to the greater percentage of patients with ischemic cardiomyopathy in this study, and may be related to the cut-off values used for defining CRT response. Due to size limitation of the study it is difficult to interpret the significance of baseline characteristic differences between groups.  We cannot rule out confounding variables that may play a role in poor response to CRT.  Suboptimal LV lead position is one potential confounding variable contributing to poor response.

## Conclusion

Anodal stimulation is a potential underrecognized and ameliorable cause of poor response to CRT. Increased awareness of this phenomenon and correction at the time of implant and follow-up may lead to a greater number of patients benefiting from CRT.

## Figures and Tables

**Table 1:**
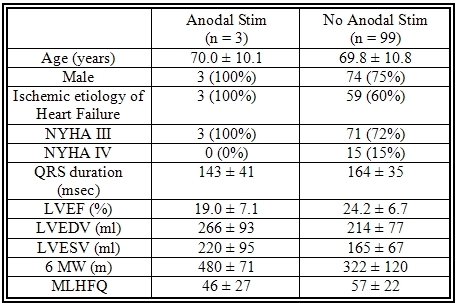
Baseline Characteristics

Values are expressed as mean ± 1 SD

**Table 2:**
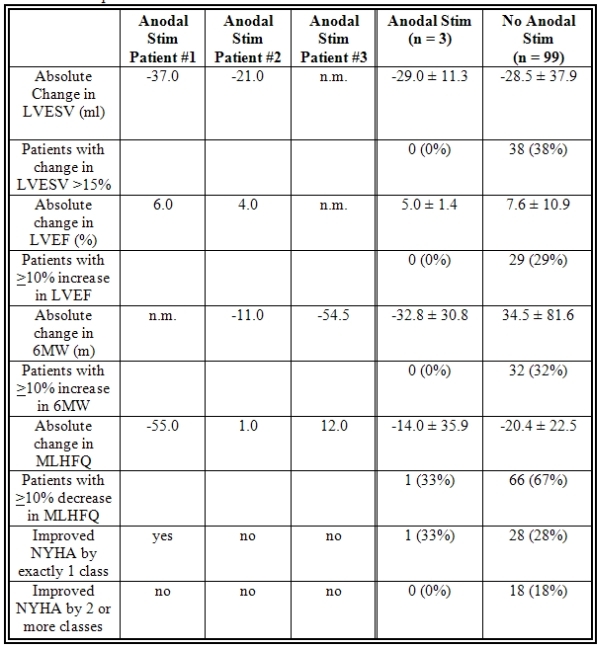
Response to CRT at six months

Values are expressed as mean ± 1 SD.  nm: not measured
